# Mesangiogenic Progenitor Cells Are Tissue Specific and Cannot Be Isolated From Adipose Tissue or Umbilical Cord Blood

**DOI:** 10.3389/fcell.2021.669381

**Published:** 2021-07-05

**Authors:** Serena Barachini, Marina Montali, Francesca M. Panvini, Vittoria Carnicelli, Gian Luca Gatti, Nicola Piolanti, Enrico Bonicoli, Michelangelo Scaglione, Gabriele Buda, Paolo D. Parchi

**Affiliations:** ^1^Department of Clinical and Experimental Medicine, University of Pisa, Pisa, Italy; ^2^Sant’Anna School of Advanced Studies, Institute of Life Sciences, Pisa, Italy; ^3^Department of Surgical, Medical and Molecular Pathology and Critical Care Medicine, University of Pisa, Pisa, Italy; ^4^Plastic and Reconstructive Surgery Unit, Azienda Ospedaliero-Universitaria Pisana, Pisa, Italy; ^5^Department of Translational Research and New Technologies in Medicine and Surgery, University of Pisa, Pisa, Italy

**Keywords:** MPCs, MSCs, bone marrow, adipose tissue, umbilical cord blood, tissue engineering, neo-vascularization

## Abstract

Mesangiogenic progenitor cells (MPCs) have been isolated from human bone marrow (BM) mononuclear cells. They attracted particular attention for the ability to differentiate into exponentially growing mesenchymal stromal cells while retaining endothelial differentiative potential. MPC power to couple mesengenesis and angiogenesis highlights their tissue regenerative potential and clinical value, with particular reference to musculoskeletal tissues regeneration. BM and adipose tissue represent the most promising adult multipotent cell sources for bone and cartilage repair, although discussion is still open on their respective profitability. Culture determinants, as well as tissues of origin, appeared to strongly affect the regenerative potential of cell preparations, making reliable methods for cell isolation and growth a prerequisite to obtain cell-based medicinal products. Our group had established a definite consistent protocol for MPC culture, and here, we present data showing MPCs to be tissue specific.

## Introduction

Mesenchymal stromal cells (MSCs), first identified in bone marrow (BM) over 50 years ago (Friedenstein et al., 1968), are characterized by their differentiative potential, both *in vitro* and *in vivo* (Caplan, 1991; Pittenger et al., 1999). Subsequent investigation focused on MSC role in repairing and healing of skeletal tissues (Jethva et al., 2009; Xiao et al., 2010), whereas, further research sparkled interest in their therapeutic potential in the regeneration of a broad spectrum of injured organs (D’souza et al., 2015). However, harvesting of BM is considered an invasive and potentially painful procedure, which also exposes donors to site morbidity (Bain, 2003). Alternative sources for MSC-like cells were considered, leading to the evidence that they could be obtained from a wide range of adult tissues and their clinical potential was investigated (Brown et al., 2019). Adipose tissue (AT) being abundant, relatively easy to access, and usually collected from discarded material after cosmetic interventions showed a valuable supply of MSCs (Zuk et al., 2001). Unlike BM, where MSCs represent a very rare population, AT can provide a high yield of cells with strong proliferative potential and therefore may be considered as a feasible source for cell therapy (Mushahary et al., 2018; Brown et al., 2019). The isolation of MSCs from AT is affected by donor’s age, health, and site of collection. In search of more primitive MSCs, fetal and perinatal tissues, including human umbilical cord blood (UCB), were also investigated (Barachini et al., 2009; Ding et al., 2015; Bieback and Netsch, 2016) and the proliferative as well as differentiating potential of derived MSCs compared (Mushahary et al., 2018). Despite a considerable amount of studies on MSC biology and clinical application, decades of efforts moving from the benchtop to the bedside have brought no consolidated MSC-based therapy (Mastrolia et al., 2019). Small variations in the isolating and culturing procedures and reagents, such as centrifugation g-force, and basal media formulation, as well as serum quality and concentration, can significantly affect the yield and composition of the isolated MSC population (Brown et al., 2019). In addition, the heterogeneity of cell culture protocols hampers a definite assessment of *in vitro*, *in vivo*, and clinical results, thus impeding confirmation of the therapeutic potential of MSC-based treatments.

Pacini suggested that the heterogeneity of MSC preparations could be considered a consequence of the combined effects of stochastic fluctuations and deterministic variations, with apparently minimal modifications of culture determinants strongly affecting cell composition and regenerative potential of cell-based medicinal products (Pacini, 2014). As a consequence, the number of contradictory results, regarding efficacy of the MSC-based therapies, could be explained by the comparisons of data produced applying significantly different cell populations, erroneously grouped under the same acronym MSCs. For instance, in 2014, Pacini hypothesized that the co-isolation of the mesangiogenic progenitor cells (MPCs), described by our group in 2009, could be responsible for the controversial data regarding the genuine angiogenic potential of MSC cultures. Although these cells can be co-isolated with MSC culture, different protocols may determine a different yield of MPCs that has been demonstrated retaining higher angiogenic potential (Pacini and Petrini, 2014). MPCs have been identified in human BM mononuclear cell (BM-MNC) cultures using autologous sera as a supplement instead of standard fetal bovine serum (Petrini et al., 2009). High-purity-grade (> 95%) MPC cultures were obtained under selective culture conditions, including medium supplementation with 10% pooled human AB-type serum (PhABS) and no gas-treated hydrophobic plastics (Trombi et al., 2009; Montali et al., 2016a). MPCs attracted particular attention for their ability to efficiently differentiate into exponentially growing MSCs, activating the Wnt5/calmodulin signaling pathway (Fazzi et al., 2011). They also retained the ability to differentiate toward the endothelial lineage. More recently, we confirmed MPC genuine angiogenic potential both *in vitro* and *in vivo*, demonstrating the mesengenic and angiogenic potentials to be mutually exclusive (Montali et al., 2017). MPCs possess longer telomeres and express pluripotency-associated markers including Oct-4 and Nanog. In particular, nestin has been considered a marker for BM-derived MPCs (Pacini et al., 2010). Cell sorting experiments showed that a highly specific BM subpopulation, described as *Pop#8* and identified by the CD64^*bright*^CD31^*bright*^CD14^*neg*^CD45^*dim*^ phenotype, represents the only BM subpopulation able to generate MPCs in culture under selective conditions (Pacini et al., 2016). MPCs’ ability to undergo dual lineage differentiation (mesengenesis vs. angiogenesis) underlines their great tissue regenerative potential and clinical value, especially in musculoskeletal tissues regeneration (Giannotti et al., 2013; Savelli et al., 2018).

With the aim of extending the range of tissue sources for MPCs herein we evaluated the efficacy of our MPC isolation and culture protocol using three candidate tissues, including BM, human stromal vascular fraction (SVF), and UCB.

## Materials and Methods

### Cell Isolation and Culture From Human BM

BM aspirates were obtained from 32 patients (16M/16F, median age = 68 years, age range = 52–85 years) undergoing orthopedic surgery for hip replacement. A 20-mL syringe containing 500 IU of heparin was used to aspirate 10 mL of BM immediately after femoral neck osteotomy during femoral reaming; the samples were collected instead of being discarded as usual, without any alteration of the standard surgical procedures. BM-MNCs were isolated and expanded as previously published. In particular, we applied the exact protocol described in 2009 (Trombi et al., 2009), validated in 2016 (Montali et al., 2016a), and described below.

Fresh BM samples were diluted 1:4 in Dulbecco’s modified phosphate-buffered saline (D-PBS; Thermo Fisher Scientific, Waltham, MA, United States) and gently layered on Ficoll-Paque^TM^ PREMIUM (GE Healthcare, Uppsala, Sweden). Samples were centrifuged at 400 g for 25 min and MNCs harvested at the interface, filtered on 70-μm filters, and washed twice in D-PBS. Cells were plated at 8 × 10^5^/cm^2^ in hydrophobic T-75 flasks (GreinerBio-One, Kremsmünster, Austria) and cultured in low-glucose Dulbecco modified Eagle medium (DMEM; Thermo Fisher Scientific) supplemented with 10% pooled human AB type serum (PhABS), 2 mM Glutamax^®^ (Thermo Fisher Scientific), and 100 μg/mL gentamicin (Thermo Fisher Scientific). PhABS batch was purchased from Lonza (Basel, Switzerland) and manufactured by the “off-the-clot” method from male sera only. The batch has been previously evaluated for its performance in MPC isolation from BM-MNCs. Validation criteria have been previously reported in Montali et al. (2016b). Culture medium was changed every 48 h. After 5–6 days, plates were morphologically screened for MPCs using an inverted microscope, cells detached by TrypLE Select^®^ (Thermo Fisher Scientific) digestion and washed in D-PBS.

### Cell Isolation and Culture From Human UCB

Donors undergoing delivery were recruited in the study. Samples were harvested from normal term pregnancies (*n* = 26) between 37 and 42 weeks of gestation, both after vaginal or cesarean section delivery. The umbilical blood was allowed to flow into heparinized tubes (5,000 IU/mL) and processed within 12 h. Samples were then diluted with D-PBS (Thermo Fisher Scientific) and MNCs collected by density gradient centrifugation using Ficoll-Paque^TM^ PREMIUM (GE Healthcare) and cultured as described above applying the protocol validated for BM-MNCs and the same PhABS batch described above.

### Cell Isolation and Culture From Human SVF

Adipose tissue was collected from patients undergoing cosmetic liposuction (*n* = 7), three from the abdominal area and four from the buttocks. In brief, 250-mL samples of liposuctioned material were extensively washed with equal volumes of D-PBS to remove erythrocytes and centrifuged for 5 min at 600 g to separate fat from oil and liquid phases. After washing, fat was combined vol/vol with 125 CDU/mL type IV collagenase (Thermo Fisher Scientific) and incubated for 1 h at 37°C in a shaking water bath. Samples were then filtered through a 100-μm filter and SVF harvested by centrifugation at 600*g* for 10 min. The resulting pellet was resuspended, and MNCs isolated and cultured under the MPC selective conditions, validated for BM-MNCs and described above taking care of applying the same PhABS batch.

After cell harvesting, cell yields have been calculated dividing absolute number of freshly detached cells by number of seeded cells, recorded as percentage (yield %) and reported as mean values ± SEM. Non-parametric Wilcoxon test for unmatched pairs was performed applying the GraphPad Prism^®^ software (GraphPad Software, San Diego, CA, United States).

### Flow Cytometry

MNCs from the three above sources (150,000 cells per sample) were incubated with REAfinity^®^ anti-human CD64 (clone REA978) fluorescein isothiocyanate–conjugated, CD31 (clone REA730) PE/Cy7-conjugated, CD14 (clone REA599) VioGreen^®^ -conjugated, and CD45 (clone REA747) VioBlue^®^ -conjugated antibodies (Miltenyi Biotec, Bergisch Gladbach, Germany) for 30’ at 4°C in the dark, and washed twice in MACS Quant^®^ Running Buffer (Miltenyi Biotec). Data were acquired using MACS Quant^®^ flow cytometer and analyzed by MACS Quantify^®^ Analysis Software (Miltenyi Biotec).

Flow cytometry of freshly detached cells from primary cultures was performed as described above using antihuman CD90 (clone DG3) FITC-conjugated, CD73 (clone AD2) PE-conjugated, CD31 PE/Cy7-conjugated, CD14 VioGreen^®^ -conjugated, and CD45 VioBlue^®^ -conjugated antibodies (Miltenyi Biotec).

Frequencies of cell populations were calculated on total events, after exclusion of cell debris on FSC vs. SSC density plots and doublets on FSC-A vs. FSC-H. Non-parametric Wilcoxon test for unmatched pairs was performed applying GraphPad Prism^®^ software (GraphPad Software, San Diego, CA, United States).

### Characterization of Cells From Primary Cultures

Cell characterization was performed according to the MPC identification protocol (Montali et al., 2016a).

#### Mesengenic Differentiation

Freshly detached cells from primary cultures were replated at 20,000 cells/cm^2^ and let adhere in DMEM/10% PhABS for 24 h. Culture medium was then replaced with StemMACS^®^ MSC Expansion Media XF (Miltenyi Biotec), and cells cultured up to 80% of confluence (usually 7–8 days) to obtain P1-MSCs. Cultures were then incubated for further 7–8 days to complete mesengenic differentiation (P2-MSCs). Cell osteogenic and adipogenic potential was tested. P2-MSCs were replated at 20,000 cells/cm^2^ in TC-treated 6-wells plates and grown to confluence. Medium was then replaced with either StemMACS^®^ OsteoDiff Media, StemMACS^®^ AdipoDiff Media, or expansion medium (negative controls). Two to 3 weeks later, calcium deposits were revealed by staining with alizarin S (Sigma Aldrich) and lipid droplets revealed by staining with Nile red 200 nM (Thermo Fisher Scientific), according to manufacturer’s. Imaging was performed on inverted fluorescence DM IRB Leica microscope (Leica, Wetzlar, Germany), equipped with LAS image acquisition software (Leica).

#### Sprouting Angiogenesis Assay

We generated a minimum of two spheroids per sample by the hanging drop method (1.5 × 10^4^ cells/spheroid). Spheroids were let to sprout out on Geltrex^®^ LDEV-free reduced growth factor basement membrane matrix (Thermo Fisher Scientific) in EGM-2 endothelial growth medium (Lonza). Spheroids were checked and imaged at 24 h and 7 days of culture, on inverted fluorescence DM IRB Leica microscope (Leica, Wetzlar, Germany), equipped with LAS image acquisition software (Leica). Quantification of sprouting distance was assessed independently by three examiners (S.B., M.M., and F.M.P.) using QWin^®^ Image Analysis software (Leica); values were reported as mean values ± SEM and two-tailed unpaired *t* test was performed.

#### Nestin Detection and F-Actin Organization Analysis

Primary cultures were grown in 2-well Lab-Tek^®^ Chamber slides. Cells were then fixed in 4% paraformaldehyde and permeabilized in 0.05% Triton X-100 for 30 min. Slides were incubated with mouse monoclonal antibody against nestin (1:150, clone 10C2, Abcam, Cambridge, United Kingdom) and after extensive washing nestin was revealed by AlexaFluor^®^ 488 Goat Anti-Mouse SFX Kit (Thermo Fisher Scientific), according to manufacturer’s. Slides were then stained with phalloidin AlexaFluor^®^ 555-conjugated antibody (Thermo Fisher Scientific) for 30 min to reveal F-actin organization. Nuclei were detected by ProLong^®^ Gold antifade reagent with 4’,6-diamidino-2-phenylindole (DAPI; Thermo Fisher Scientific).

### Gene Expression Profile of Cells From Primary Cultures

Gene expression analysis was performed on cells from five primary cultures for each of the three different tissue sources. Custom 96-well PrimePCR^®^ Plates (BioRad, Hercules, CA, United States) including primer sets for 87 target genes, 5 reference genes (Supplementary Table 1), and 5 internal controls were used for gene expression profiling of P1-MSCs. Total RNAs were purified from freshly detached cells using Direct-zol RNA MicroPrep Kit (Zymo Research, Irvine, CA, United States) and quantified with Qubit 4 Fluorometer (Thermo Fisher Scientific) by Qubit RNA HS Assay Kit (Thermo Fisher Scientific). cDNAs were synthesized from 1 μg of total RNA using iScript gDNA Clear cDNA Synthesis Kit, according to manufacturers. Quantitative polymerase chain reaction (qPCR) was carried out with SsoAdvanced Unversal SybrGreen Supermix (BioRad), on iQ5 Real-Time PCR Detection System (BioRad), according to PrimePCR Array^®^ instruction manual. Fold changes calculation by ΔΔC_*t*_ method and statistical analysis were assessed by PrimePCR^®^ Analysis software (BioRad). According to the manufacturer, the *p* values reported on the results table are the result of unpaired *t* tests comparing the distributions of per well normalized expression (NE) values for the control sample (BM-MNCs) versus the test sample (UCB-MNCs). C_*t*_ values higher than 35, were considered as “no expression.” After the analysis of the relative stability, two reference genes (*B2M*, *GAPDH*) were validated for normalization.

## Results

### Flow Cytometry Quantification of MPC *in vivo* Progenitors (Pop#8)

**FIGURE 1:**
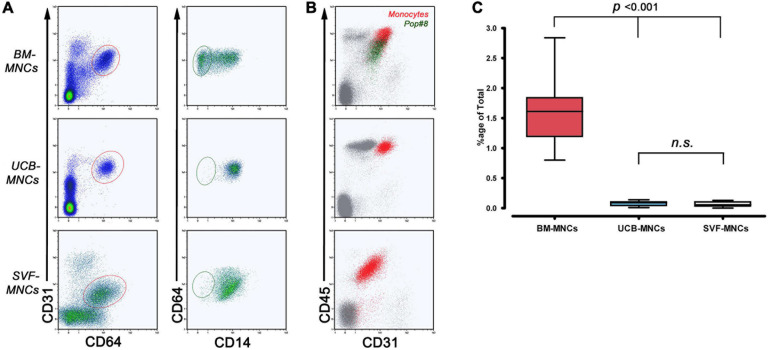
Flow cytometry quantification of MPC in vivo progenitors (Pop#8). **(A)** To quantify Pop#8 population in the three different tissue sources, the specific gating strategy has been applied. In details, CD64^*bright*^CD31^*bright*^ events (elliptical region in red) were displayed on CD64 vs. CD14 density plots in order to quantify the CD64^*bright*^CD31^*bright*^CD14^*neg*^ population (elliptical region in dark green), representing the genuine in vivo progenitor of the MPCs (Pop#8). This population was consistently detected in BM-MNCs only. **(B)** CD45 vs. CD31 dot plots confirmed the characteristic CD45 dim expression on Pop#8 (dark green dots) in contrast to the bright expression on CD64^*bright*^CD31^*bright*^CD14^+^ monocytes (red dots). **(C)** Mean percentage of Pop#8 in BM-MNCs resulted in approximately 1.5% of the total. n.s., not significant.

We used multicolor flow cytometry to identify and quantify *Pop#8* MPC *in vivo* progenitors in freshly isolated MNCs from BM-MNCs, UCB-MNCs, and SVF-MNCs. The *Pop#8* immunophenotype was previously described as CD64^*bright*^CD31^*bright*^CD14^*neg*^CD45^*dim*^ (Figures 1A,B; Pacini et al., 2016). A CD64^*bright*^CD31^*bright*^ subpopulation was clearly detectable in both BM- and UCB-MNCs while SVF-MNCs expressed lower levels of CD31. However, we identified the genuine *Pop#8* immunophenotype defined as CD14^*neg*^CD45^*dim*^ in BM-MNCs only and quantification revealed consistent to previous results (1.60% ± 0.12%, Figure 1C and Supplementary Table 2). In UCB-MNCs almost the entire CD64^*bright*^CD31^*bright*^ population was represented by CD14-positive mature monocytes (red dots in Figure 1B). In SVF-MNCs the CD64/CD31-positive population expressed CD14 and CD45 although at lower intensities. Differences in expression could be ascribed to SVF-MNC different isolating procedure.

### Morphology, Immunophenotype, and Yield of Cells From Primary Cultures Under MPC Selective Conditions

**FIGURE 2:**
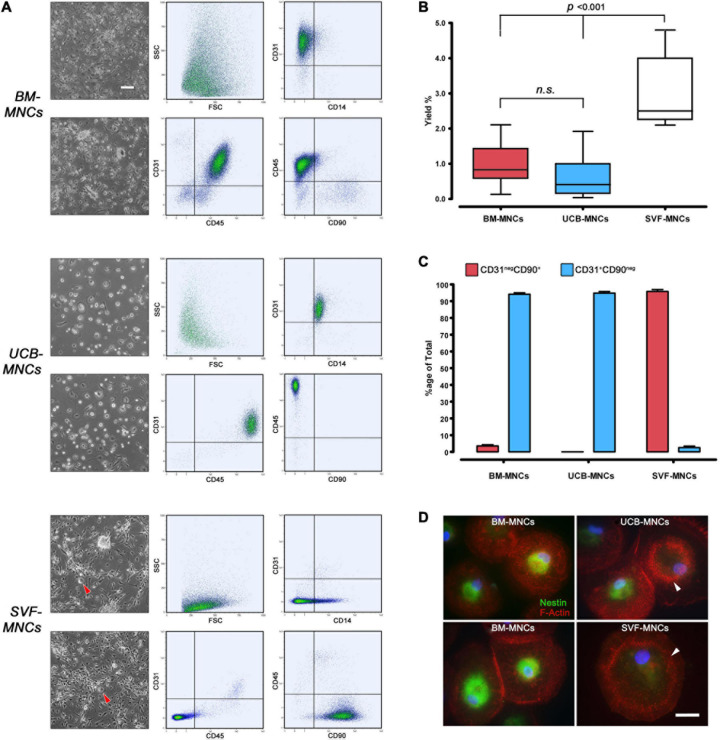
Primary culture under MPC selective culture conditions. **(A)** After a week of culture in DMEM/10% PhABS on hydrophobic plastics, adherent cells from BM- and UCB-MNCs showed similar rounded refringent morphology. However, cells from UCB-MNCs appeared larger and flattened, with very rare interspersed polar elongated cells frequently detected in BM-MNC cultures. SVF-MNC cultures resulted in an almost confluent layer of fibroblastoid MSC-like cells with sporadic rounded refringent cells (red arrowheads). CD31^+^CD45^*dim*^CD14^*neg*^CD90^*neg*^ phenotype of BM-MNC culture generated cells was distinctive of MPCs at a difference with the macrophagic CD14^+^CD45^*bright*^CD31^+^phenotype displayed by most UCB-derived cells. **(B)** Cell recovery was significantly higher from SVF-MNCs possibly due to the expansion of proliferating cells similar to MSCs as demonstrated by their CD31^*neg*^CD90^+^ phenotype (**C**, red bars). A very small population of MSC-like cells was detected also in BM-derived cultures. **(D)** Nestin (green) was found in the vast majority of cells from BM-MNCs, showing dispersed podosomes (red). Most cells from UCB-MNCs were nestin-negative and characterized by a “belt” distribution of podosomes, similarly to the rare rounded cells detected in SVF cultures. n.s., not significant.

After 5–6 days of culture under MPC selective conditions, BM-MNCs generated rounded, highly refringent, firmly attached cells. Their high side scatter (SSC) signal and CD14^*neg*^CD45^*dim*^CD31^+^ phenotype, lacking MSC-related antigens CD90 and CD73, allowed us to identify them as MPCs. UCB-MNC cultures generated fewer larger cells that, despite the MPC-like morphology, were identified as macrophages because of their CD14^+^CD45^*bright*^CD31^+^ phenotype. The spindle-shaped morphology and CD90^+^CD73^+^ phenotype of SVF-MNC–derived cells were reminiscent of standard AT-MSCs (Figure 2A). Very rare CD14^+^CD31^+^CD45^+^ rounded cells were also detected (Figure 2A, red arrowheads). MPC yield from BM-MNCs was consistent with previous data (0.97% ± 0.12%), while yield from UCB-MNCs was slightly lower (0.63% ± 0.13%). Significantly higher yield was evidenced from SVF-MNC cultures (3.02% ± 0.38%, *p* < 0.001) probably due to the proliferating nature of the MSC-like cells (Figure 2B), which represent more than 95% of cell population. Consistent with previous reports, a small population of MSC-like cells (3.51% ± 0.78%) was also detected in BM-MNC cultures, at a difference with UCB-MNCs (Figure 2C).

Most cells from BM-MNC primary cultures expressed nestin and showed dispersed podosome-like structures as revealed by F-actin dotted pattern of expression, characteristic of MPC phenotype (Pacini et al., 2013). A significant number of nestin-negative cells, showing “belt” distribution of podosomes, were detected in UCB-MNC cultures. The rare rounded cells co-isolated in SVF-MNC cultures were all nestin-negative and showed the “belt” podosome pattern (Figure 2D, white arrowheads).

### Differentiation Potential of Cells From Primary Cultures

**FIGURE 3:**
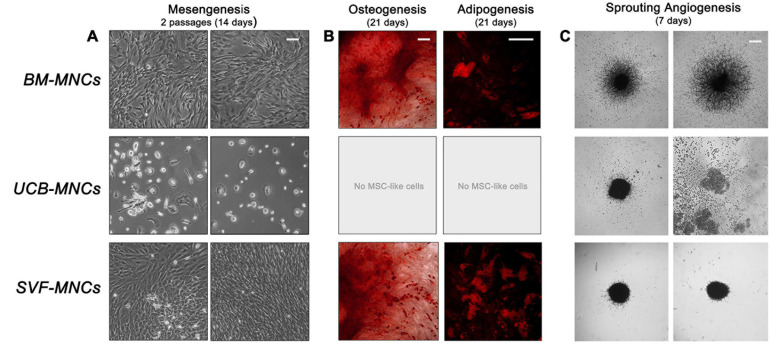
Mesangiogenic potential of cells from primary cultures. **(A)** After 2 weeks of mesengenic induction the rounded refringent MPCs from BM-MNCs differentiated into proliferating fibroblastoid MSCs. Conversely, cells from UCB-MNCs maintained their morphology with no sign of differentiation. Spindle-shaped cells from SVF-MNC primary cultures could be expanded with unaltered morphology. **(B)** Terminal osteogenic or adipogenic differentiation confirmed the MSC-like nature of the cells from BM and SVF after mesengenic induction. **(C)** Only BM-derived MPCs showed a consistent sprouting activity under angiogenic stimulus, confirming their mesangiogenic potential.

We analyzed the mesengenic potential of cells from primary cultures by the two step protocol previously described (Fazzi et al., 2011). We were able to obtain P2-MSCs from BM- and SVF-MNC primary cultures, whereas, cells isolated from UCB-MNCs failed to differentiate. They kept their round morphology and did not proliferate at all, notwithstanding the 14-day culture in differentiating conditions (Figure 3A). The MSC nature of BM- and SVF-derived P2-MSCs was definitely demonstrated by their terminal differentiation into either osteoblasts or adipocytes. After further 3 weeks of culture under osteogenic or adipogenic induction, extracellular calcium deposits, and intracellular lipid droplet accumulation were revealed by alizarin S and Nile red stains, respectively (Figure 3B).

Sprouting angiogenesis assay revealed that only MPCs from BM-MNCs retained angiogenic potential with more than 300 μm sprouting from 3D spheroids (325.1 ± 29.9 μm). Cells from UCB-MNCs gave origin to few loose cell aggregates, which lacked the mechanical properties required for handling. As a consequence, the spheroids disaggregated during seeding, and no sign of ECM degradation was reported. Compact spheroids were obtained from AT-derived cells without evidence of significant sprouting activity (27.8 ± 9.1 μm, *p* < 0.0001), under vascular endothelial growth factor (VEGF) stimulus (Figure 3C).

### Gene Expression Profile of Cells From Primary Cultures

**FIGURE 4:**
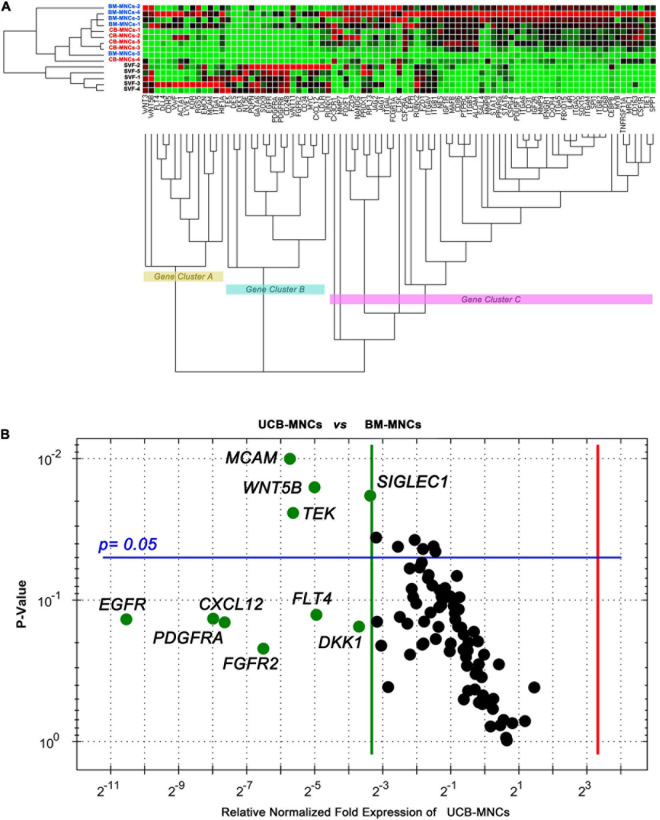
Gene expression profiling of cells from primary cultures. **(A)** BM- and UCB-derived cells showed parallel expression profiles for the 87 genes analyzed. Angiogenesis/lymphangiogenesis (gene cluster A) and MPC-related genes (gene cluster C) were significantly up-regulated in these cells, whereas, MSC-related genes (cluster B) resulted up-regulated in SVF-derived cells. **(B)** Single-gene expression analysis revealed significant lower expression of MCAM, WNT5B, TEK, and SIGLEC1 in UCB-derived cells as compared to BM-derived cells. Relevant reduction of stromal growth factor receptor gene expression was also detected.

Unsupervised cluster expression analysis of 87 target genes in cells from primary cultures revealed three main clusters (gene clusters A–C in Figure 4A). Cluster A included a number of angiogenesis- and lymphoangiogenesis-associated genes (*FLT4*, *LYVE1*, *DLL4*, *KDR*, *VWF*, and *EMCN)* as well as pericyte markers *(RGS5* and *MCAM)*. Cluster B included MSC-related genes (*DES*, *DKK1*, *NT5E*, *SOX9*, *EGFR*, and *PDGFR*), while most genes in cluster C were associated to MPCs (*SPP1*, *ITGB2*, *SOX15*, and *FBX15*, in particular). Cells derived from BM- and UCB-MNC cultures showed increased expression of gene cluster C and reduced expression of gene cluster B. Conversely, gene expression profile of SVF-MNCs was characterized by up-regulation of cluster B and down-regulation of cluster C. Comparison of single-gene expression between BM- and UCB-MNCs revealed substantially lower levels of some genes of interest, in the latter. In particular, *MCAM* reduction was approximately 50-fold (0.0394 ± 0.0117 vs. 2.0903 ± 1.8011, *n* = 5), *WNT5B* approximately 30-fold (0.0306 ± 0.0348 vs. 0.9842 ± 1.0254, *n* = 5), *TEK* almost 50-fold (0.0069 ± 0.00743 vs. 0.3461 ± 0.2615, *n* = 5), and *SIGLEC1* close to 10-fold (0.0943 ± 0.0301 vs. 0.7012 ± 0.2243, *n* = 5, Figure 4B). Drastic reduction in the expression of EGF (-1,486.4), PDGF (-253.6), FGF-2 (-91.2), VEGF (-30.9) receptor genes, and *CXCL12* (-200.6) was also detected in UCB-MNCs, although data were too variable for statistical significance.

## Discussion

MSCs and their *in vivo* ancestors hold great promise for the treatment of bone and cartilage defects (Lin et al., 2017) as shown by their ability to enhance bone repair in a wide range of animal model systems (Pacini et al., 2007; Jafarian et al., 2008; De Schauwer et al., 2013). To date, BM-MNCs and AT-derived SVF are still the main sources of adult multipotent cells for autologous cell–based therapies (Hoogduijn and Dor, 2013; Shariatzadeh et al., 2019). Both BM- and AT-MSCs have been used to repair various bone defects (Marcacci et al., 2007; Mesimaki et al., 2009; Gimble et al., 2010; Lindroos et al., 2011). Regeneration of articular cartilage has been achieved by applying both BM- and AT-MSC in models of osteochondral defect (Ishihara et al., 2014; Murata et al., 2015; Itokazu et al., 2016; Li et al., 2018).

Bone marrow- and AT-MSCs share a number of features, including morphology and cell surface markers. However, significant biological differences have been found in their proliferation/differentiation properties (Danisovic et al., 2009), and the discussion on their respective regenerative potential is still open (Huang et al., 2005; Elman et al., 2014; Rasmussen et al., 2014). Despite remarkable improvements in isolation, expansion, and characterization of adult multipotent cells, clinical and preclinical trials often showed disappointing outcomes with lack of efficacy in long-lasting consolidated repair (Mastrolia et al., 2019; Shariatzadeh et al., 2019). A primary reason of such unsatisfactory results could be lack of or inefficient vascularization in newly formed tissues (Chung and Shum-Tim, 2012).

Nonetheless, BM-MSCs still represent the most applied cells for the engineering of cell-based medicinal products (CBMPs) (Mastrolia et al., 2019), with a number of preclinical studies showing BM-derived cells to be more effective in the regeneration and repair of skeletal tissues than alternative sources (Brennan et al., 2017). AT-MSCs demonstrated inferior *in vivo* osteogensis and superior angiogenesis as compared to BM stromal cells (Brennan et al., 2017), casting doubts on AT-MSC use in bone repair because of their limited osteogenic differentiation potential. In the present study we showed AT-MSCs not to possess intrinsic vasculogenic potential, corroborating the idea that their contribution to new vessel formation would be exerted exclusively by the secretion of specific angiogenic factors. Thus, vascularization of AT-MSC engineered implants strictly depends on perfusion of the surrounding microenvirorment. This represents a further limiting factor in regenerating naturally low vascularized tissues, as bone and cartilage, or compromised injured sites as non-union fractures.

Our results demonstrated that MPCs are tissue specific and, in accordance with what previously reported (Montali et al., 2016a, 2017), CD64^*bright*^CD31^*bright*^CD14^*neg*^CD45^*dim*^ *Pop#8* MPC progenitors were consistently detected exclusively in BM-MNCs leading to the isolation of MPCs under selective culture conditions. Extended *Pop#8* characterization revealed CD45 to be mildly expressed while most of the antigens feasible for prospective isolation of MSCs from BM remained unexpressed (Pacini et al., 2016). In particular, the lack of both CD146 and CD271 expression suggests that *Pop#8* should be considered distinct from the CD146^*bright*^ pericytes found in the subendothelial layer of sinusoids (Sacchetti et al., 2007), from the trabecular bone-lining CD271^+^CD146^*neg*^ cell population (Tormin et al., 2011) and from the stromal reticular cells as well (Omatsu et al., 2010), all of them described as *in vivo* MSC progenitors in the BM, sustaining the idea of a multiple origin of MSCs. Interestingly, similar CD146^*bright*^ perivascular cell population has been found in SVF from the AT (Crisan et al., 2008; Corselli et al., 2012) suggesting that BM and AT could share a common perivascular progenitor for the MSCs, whereas, *Pop#8* is exclusively detected in BM and at significantly higher frequency respect to pericytes.

Here, we hypothesize that BM concentrates and BM-MSC superior performances in skeletal tissue regeneration, could be explained by the presence of MPCs and/or *Pop#8* progenitors. Their essential chondrogenic and osteogenic potential would couple with their capability to trigger new blood vessel formation in implant early phases. Interestingly, specific endothelial cells were found in tight relation with chondrocytes and osteogeoprogenitors in the growth plate of long developing bones (Kusumbe et al., 2014). According to the “developmental engineering” paradigm (Lenas et al., 2009), vascularization is vital to bone tissue regeneration, and conception of new CBMPs should take it into consideration. Researchers and clinical community rely on the increasing knowledge of angiogenic and vasculogenic processes stimulating a clinically relevant vascular network formation within the implanted engineered constructs. In this view, clinical application of MPC-based CBMPs could take advantage from the unique features of these adult multipotent cells. MPCs are found at frequencies from one to two logs higher than other BM-MSC progenitors and vast numbers could be readily isolated in 4–6 days from 10 to 15 mL of fresh BM using a cheap GMP-compliant culture method (Montali et al., 2016a). The lack of requirement for *in vitro* cell expansion minimizes culture times and carries significant advantages in terms of reduced risk of cell transformation, cellular senescence, and exposition to bacterial and viral contamination. Moreover, the application of undifferentiated MPCs could also provide beneficial effects on producing functional long-lasting healing of target tissues.

## Data Availability Statement

All relevant data is contained within the article. The original contributions presented in the study are included in the article/Supplementary Material, further inquiries can be directed to the corresponding author. Moreover, datasets are available on request and the raw data supporting the conclusion of this article will be made available by the authors, without undue reservation.

## Ethics Statement

The studies involving human participants were reviewedand approved by Ethics Committee of *AziendaOspedaliero-Universitaria Pisana* – *Comitato Etico di Area Vasta Nord Ovest* (CEAVNO) (committee approval number: 48812/07). The patients/participants provided their written informed consent to participate in this study.

## Author Contributions

SB and MM were responsible for the conception and design, acquisition, analysis, interpretation of data, and drafting the article. FMP was responsible for acquisition and analysis of data. VC was responsible for qPCR data acquisition. GLG was responsible for adipose tissue sample collection. NP and EB were responsible for bone marrow sample collection. MS and GB have critically revised the manuscript. PP was responsible for bone marrow sample collection and approved the final version to be published. All authors read and approved the final manuscript.

## Conflict of Interest

The authors declare that the research was conducted in the absence of any commercial or financial relationships that could be construed as a potential conflict of interest.
