# Supplementary material for: Mesangiogenic Progenitor Cells Are Tissue Specific and Cannot Be Isolated From Adipose Tissue or Umbilical Cord Blood
Source: Front Cell Dev Biol. 2021 Jul 5;9:669381. doi: 10.3389/fcell.2021.669381 (PMC8287027; doi:10.3389/fcell.2021.669381)
Supplement: Supplementary file 1 [file Table_1.pdf]

Supplementary Table S1. *Custom 96-well PrimePCR™ Plates, gene list*

Well	Gene Name	Gene Symbol	Entrez Gene ID	Unique Assay Id	Amplicon Length (bp)
A1	actin, alpha 1, skeletal muscle	ACTA1	58	qHsaCED0045151	60
A2	cadherin 5, type 2 (vascular endothelium)	CDH5	1003	qHsaCID0016288	112
A3	chemokine (C-X-C motif) receptor 4	CXCR4	7852	qHsaCED0002020	142
A4	fibroblast growth factor receptor 2	FGFR2	2263	qHsaCID0021928	124
A5	insulin-like growth factor 1 receptor	IGF1R	3480	qHsaCID0015667	81
A6	integrin, alpha V (vitronectin receptor, alpha polypeptide, antigen CD51)	ITGAV	3685	qHsaCID0006233	130
A7	low density lipoprotein receptor	LDLR	3949	qHsaCID0015114	121
A8	mannose receptor, C type 1	MRC1	4360	qHsaCID0037074	77
A9	podoplanin	PDPN	10630	qHsaCID0009013	134
A10	sal-like 4 (Drosophila)	SALL4	57167	qHsaCID0014611	157
A11	tyrosine kinase with immunoglobulin-like and EGF-like domains 1	TIE1	7075	qHsaCID0006540	75
A12	glyceraldehyde-3-phosphate dehydrogenase	GAPDH	2597	qHsaCED0038674	117
B1	activated leukocyte cell adhesion molecule	ALCAM	214	qHsaCID0037887	173
B2	CCAAT/enhancer binding protein (C/EBP), beta	CEBPB	1051	qHsaCED0019041	117
B3	desmin	DES	1674	qHsaCID0011768	116
B4	fms-related tyrosine kinase 4	FLT4	2324	qHsaCID0020886	150
B5	insulin-like growth factor 2 receptor	IGF2R	3482	qHsaCID0018295	112
B6	integrin, alpha X (complement component 3 receptor 4 subunit)	ITGAX	3687	qHsaCID0006223	104
B7	leptin receptor	LEPR	3953	qHsaCID0018270	131
B8	v-myb myeloblastosis viral oncogene homolog (avian)	MYB	4602	qHsaCED0043781	105
B9	POU class 5 homeobox 1	POU5F1	5460	qHsaCED0038334	100
B10	SRY (sex determining region Y)-box 15	SOX15	6665	qHsaCED0044255	78
B11	tumor necrosis factor receptor superfamily, member 11a, NFKB activator	TNFRSF11A	8792	qHsaCID0006213	99
B12	hypoxanthine phosphoribosyltransferase 1	HPRT1	3251	qHsaCID0016375	90
C1	platelet and endothelial cell adhesion molecule 1	PECAM1	5175	*	231
C2	colony stimulating factor 1 receptor	CSF1R	1436	qHsaCID0010604	150
C3	dickkopf homolog 1 (Xenopus laevis)	DKK1	22943	qHsaCED0002060	118
C4	forkhead box F1	FOXF1	2294	qHsaCED0002932	133
C5	interleukin 4 receptor	IL4R	3566	qHsaCID0008648	143
C6	integrin, beta 1 (fibronectin receptor, beta polypeptide, antigen CD29 includes MDF2, MSK12)	ITGB1	3688	qHsaCED0005248	104
C7	lymphatic vessel endothelial hyaluronan receptor 1	LYVE1	10894	qHsaCID0010430	126
C8	v-myc myelocytomatosis viral oncogene homolog (avian)	MYC	4609	qHsaCID0012921	103
C9	peroxisome proliferator-activated receptor gamma	PPARG	5468	qHsaCID0011718	117
C10	SRY (sex determining region Y)-box 9	SOX9	6662	qHsaCED0021217	77
C11	von Willebrand factor	VWF	7450	qHsaCED0033955	113
C12	ribosomal protein L13	RPL13	6137	qHsaCED0056592	114
D1	CD163 molecule	CD163	9332	qHsaCID0012333	150
D2	colony stimulating factor 2 receptor, alpha, low-affinity (granulocyte-macrophage)	CSF2RA	1438	qHsaCID0013436	92
D3	delta-like 4 (Drosophila)	DLL4	54567	qHsaCID0008450	84
D4	frizzled homolog 1 (Drosophila)	FZD1	8321	qHsaCED0018783	116
D5	integrin, alpha 1	ITGA1	3672	qHsaCID0017712	144
D6	integrin, beta 2 (complement component 3 receptor 3 and 4 subunit)	ITGB2	3689	qHsaCED0003958	63
D7	v-maf musculoaponeurotic fibrosarcoma oncogene homolog B (avian)	MAFB	9935	qHsaCED0002199	115
D8	Nanog homeobox	NANOG	79923	qHsaCED0043394	158
D9	prospero homeobox 1	PROX1	5629	qHsaCID0018340	86
D10	spleen focus forming virus (SFFV) proviral integration oncogene spi1	SPI1	6688	qHsaCID0022097	96
D11	wingless-type MMTV integration site family, member 11	WNT11	7481	qHsaCID0011927	88
D12	PrimePCR DNA Contamination Control Assay	gDNA	--	qHsaCtlD0001004	--
E1	CD248 molecule, endosialin	CD248	57124	qHsaCED0018682	121
E2	chondroitin sulfate proteoglycan 4	CSPG4	1464	qHsaCID0005989	148
E3	epidermal growth factor receptor	EGFR	1956	qHsaCID0007564	97
E4	frizzled homolog 9 (Drosophila)	FZD9	8326	qHsaCED0019089	69
E5	integrin, alpha 5 (fibronectin receptor, alpha polypeptide)	ITGA5	3678	qHsaCID0021495	140
E6	integrin, beta 5	ITGB5	3693	qHsaCID0007523	76
E7	melanoma cell adhesion molecule	MCAM	4162	qHsaCID0020233	146
E8	nestin	NES	10763	qHsaCED0001303	87
E9	protein tyrosine phosphatase, receptor type, C	PTPRC	5788	qHsaCED0038908	69
E10	secreted phosphoprotein 1	SPP1	6696	qHsaCID0012060	99
E11	wingless-type MMTV integration site family, member 3	WNT3	7473	qHsaCID0015193	103
E12	PrimePCR Positive Control Assay	PCR	--	qHsaCtlD0001003	--
F1	CD34 molecule	CD34	947	qHsaCID0007456	99
F2	cathepsin K	CTSK	1513	qHsaCID0016934	147
F3	endomucin	EMCN	51705	qHsaCED0046161	91
F4	GATA binding protein 6	GATA6	2627	qHsaCED0045017	81
F5	integrin, alpha 6	ITGA6	3655	qHsaCID0007188	130
F6	jagged 1	JAG1	182	qHsaCID0006831	99
F7	matrix metallopeptidase 7 (matrilysin, uterine)	MMP7	4316	qHsaCID0011537	138
F8	5'-nucleotidase, ecto (CD73)	NT5E	4907	qHsaCID0036556	117
F9	regulator of G-protein signaling 5	RGS5	8490	qHsaCED0045107	67
F10	signal transducer and activator of transcription 1, 91kDa	STAT1	6772	qHsaCID0007580	92
F11	wingless-type MMTV integration site family, member 5B	WNT5B	81029	qHsaCID0038673	69
F12	PrimePCR RNA Quality Assay	RQ1	--	qHsaCtlD0001002	--
G1	CD68 molecule	CD68	968	qHsaCED0007025	82
G2	chemokine (C-X3-C motif) receptor 1	CX3CR1	1524	qHsaCED0046543	68
G3	F-box protein 15	FBXO15	201456	qHsaCID0021364	144
G4	hypoxia inducible factor 1, alpha subunit (basic helix-loop-helix transcription factor)	HIF1A	3091	qHsaCID0014755	72
G5	integrin, alpha L (antigen CD11A (p180), lymphocyte function-associated antigen 1; alpha polypeptide)	ITGAL	3683	qHsaCID0015523	146
G6	jagged 2	JAG2	3714	qHsaCED0003193	127
G7	matrix metallopeptidase 8 (neutrophil collagenase)	MMP8	4317	qHsaCID0023232	135

G8	platelet-derived growth factor receptor, alpha polypeptide	PDGFRA	5156	qHsaCID0007202	103
G9	runt-related transcription factor 1	RUNX1	861	qHsaCID0037818	74
G10	signal transducer and activator of transcription 6, interleukin-4 induced	STAT6	6778	qHsaCED0056844	120
G11	actin, beta	ACTB	60	qHsaCED0036269	62
G12	PrimePCR RNA Quality Assay	RQ2	--	qHsaCtID0001002	--
H1	CD86 molecule	CD86	942	qHsaCID0021122	97
H2	chemokine (C-X-C motif) ligand 12	CXCL12	6387	qHsaCID0012398	94
H3	Fc fragment of IgG, high affinity Ia, receptor (CD64)	FCGR1A	2209	qHsaCED0034082	144
H4	interferon gamma receptor 1	IFNGR1	3459	qHsaCID0013339	84
H5	integrin, alpha M (complement component 3 receptor 3 subunit)	ITGAM	3684	qHsaCID0006023	146
H6	kinase insert domain receptor (a type III receptor tyrosine kinase)	KDR	3791	qHsaCID0006310	140
H7	matrix metallopeptidase 9 (gelatinase B, 92kDa gelatinase, 92kDa type IV collagenase)	MMP9	4318	qHsaCID0011597	82
H8	platelet-derived growth factor receptor, beta polypeptide	PDGFRB	5159	qHsaCID0013272	117
H9	runt-related transcription factor 2	RUNX2	860	qHsaCED0044067	83
H10	TEK tyrosine kinase, endothelial	TEK	7010	qHsaCID0015119	101
H11	beta-2-microglobulin	B2M	567	qHsaCID0015347	123
H12	PrimePCR Reverse Transcription Control Assay	RT	--	qHsaCtID0001001	--

(*) Custom designed primer pair Forward Primer: GAACCTGTCCTGCTCCATC
Reverse Primer: TCAAACTGGGCATCATAAGAAAT
