# Supplementary material for: Mesangiogenic Progenitor Cells Are Tissue Specific and Cannot Be Isolated From Adipose Tissue or Umbilical Cord Blood
Source: Front Cell Dev Biol. 2021 Jul 5;9:669381. doi: 10.3389/fcell.2021.669381 (PMC8287027; doi:10.3389/fcell.2021.669381)
Supplement: Supplementary file 2 [file Table_2.pdf]

Supplemetary Table S2. *Flow Cytometry quantification of Pop#8*

	BM-MNCs			UCB-MNCs			SVF	
SampleID	**Pop#8%**	**Culture Yield%**	*SampleID*	**Pop#8%**	**Culture Yield%**	*SampleID*	**Pop#8%**	**Culture Yield%**
BM001	1,71	2,56	UCB01	0,01	0,50	SVF01	0,03	4,80
BM002	1,85	2,22	UCB02	0,02	1,61	SVF02	0,08	4,00
BM003	2,84	0,58	UCB03	0,02	1,00	SVF03	0,03	2,25
BM004	2,50	0,20	UCB04	0,02	0,09	SVF04	0,09	2,50
BM005	1,24	0,13	UCB05	0,10	0,65	SVF05	0,03	3,10
BM006	0,80	0,73	UCB06	0,09	1,37	SVF06	0,00	2,10
BM007	1,18	1,37	UCB07	0,04	1,72	SVF07	0,03	2,40
BM008	1,50	0,78	UCB08	0,07	0,08			
BM009	1,78	2,11	UCB09	0,10	0,86			
BM010	1,70	1,51	UCB10	0,05	0,00			
BM011	1,73	0,83	UCB11	0,09	0,33			
BM012	0,75	0,61	UCB12	0,10	0,04			
BM013	1,83	1,38	UCB13	0,09	0,30			
BM014	0,65	0,34	UCB14	0,06	0,83			
BM015	1,28	0,13	UCB15	0,01	0,17			
BM016	1,42	0,98	UCB16	0,07	0,06			
BM017	1,94	0,50	UCB17	0,01	0,12			
BM018	1,51	0,54	UCB18	0,07	0,41			
BM019	1,73	0,63	UCB19	0,02	1,00			
BM020	1,45	0,94	UCB20	0,00	0,35			
BM021	1,55	1,36	UCB21	0,03	0,15			
BM022	1,10	0,79	UCB22	0,01	1,80			
BM023	2,20	1,33	UCB23	0,01	1,13			
BM024	1,76	1,01	UCB24	0,02	0,60			
BM025	1,20	0,78	UCB25	0,01	0,42			
BM026	2,10	1,12	UCB26	0,03	0,87			
BM027	1,70	0,94						
BM028	1,87	0,87						
BM029	1,82	1,21						
BM030	1,87	1,10						
BM031	0,61	0,55						
BM032	1,91	0,78						
Mean	**1,60**	**0,97**	*Mean*	**0,04**	**0,63**	*Mean*	**0,04**	**3,02**
SEM	0,12	0,12	*SEM*	0,04	0,13	*SEM*	0,40	0,38
